# Comparison of FACS and PCR for Detection of BCMA-CAR-T Cells

**DOI:** 10.3390/ijms23020903

**Published:** 2022-01-14

**Authors:** Avinoam Reichman, Alexander Kunz, Jara J. Joedicke, Uta E. Höpken, Anna Keib, Brigitte Neuber, David Sedloev, Lei Wang, Genqiao Jiang, Angela Hückelhoven-Krauss, Franziska Eberhardt, Carsten Müller-Tidow, Martin Wermke, Armin Rehm, Michael Schmitt, Anita Schmitt

**Affiliations:** 1 Department of Hematology, Oncology and Rheumatology, Heidelberg University Hospital, Im Neuenheimer Feld 410, 69120 Heidelberg, Germany; avinoam.reichman@gmail.com (A.R.); alexander.kunz@med.uni-heidelberg.de (A.K.); anna.keib@med.uni-heidelberg.de (A.K.); Brigitte.Neuber@med.uni-heidelberg.de (B.N.); David.Sedloev@med.uni-heidelberg.de (D.S.); Lei.Wang@med.uni-heidelberg.de (L.W.); genqiao.jiang@med.uni-heidelberg.de (G.J.); Angela.Hueckelhoven-Krauss@med.uni-heidelberg.de (A.H.-K.); franziska.eberhardt@med.uni-heidelberg.de (F.E.); carsten.mueller-tidow@med.uni-heidelberg.de (C.M.-T.); michael.schmitt@med.uni-heidelberg.de (M.S.); 2 Department of Microenvironmental Regulation in Autoimmunity and Cancer, Max-Delbrück Center for Molecular Medicine (MDC), Robert-Rössle-Str. 10, 13125 Berlin-Buch, Germany; Jara.Joedicke@mdc-berlin.de (J.J.J.); arehm@mdc-berlin.de (A.R.); 3 Department of Translational Tumor Immunology, Max-Delbrück Center for Molecular Medicine (MDC), Robert-Rössle-Str. 10, 13125 Berlin-Buch, Germany; uhoepken@mdc-berlin.de; 4 NCT/UCC Early Clinical Trial Unit (ECTU), Medical Faculty C.-G. Carus, Technical University Dresden, Fetscherstraße 74, 01307 Dresden, Germany; martin.wermke@uniklinikum-dresden.de

**Keywords:** BCMA-CAR, polymerase chain reaction, detection reagent, flow cytometry

## Abstract

Chimeric-antigen-receptor (CAR)-T-cell therapy is already widely used to treat patients who are relapsed or refractory to chemotherapy, antibodies, or stem-cell transplantation. Multiple myeloma still constitutes an incurable disease. CAR-T-cell therapy that targets BCMA (B-cell maturation antigen) is currently revolutionizing the treatment of those patients. To monitor and improve treatment outcomes, methods to detect CAR-T cells in human peripheral blood are highly desirable. In this study, three different detection reagents for staining BCMA-CAR-T cells by flow cytometry were compared. Moreover, a quantitative polymerase chain reaction (qPCR) to detect BCMA-CAR-T cells was established. By applying a cell-titration experiment of BCMA-CAR-T cells, both methods were compared head-to-head. In flow-cytometric analysis, the detection reagents used in this study could all detect BCMA-CAR-T cells at a similar level. The results of false-positive background staining differed as follows (standard deviation): the BCMA-detection reagent used on the control revealed a background staining of 0.04% (±0.02%), for the PE-labeled human BCMA peptide it was 0.25% (±0.06%) and for the polyclonal anti-human IgG antibody it was 7.2% (±9.2%). The ability to detect BCMA-CAR-T cells down to a concentration of 0.4% was similar for qPCR and flow cytometry. The qPCR could detect even lower concentrations (0.02–0.01%). In summary, BCMA-CAR-T-cell monitoring can be reliably performed by both flow cytometry and qPCR. In flow cytometry, reagents with low background staining should be preferred.

## 1. Introduction

Chimeric-antigen-receptor (CAR)-T-cell therapies directed against the CD19 antigen are already an inherent part of the therapeutical armamentarium for defeating hematologic malignancies. There are four FDA-approved CD19-CAR-T-cell agents: Kymriah™ (tisa-cel) for the treatment of acute lymphoblastic leukemia in children and young adults [1], Yescarta™ (axi-cel) [2] and Breyanzi™ (liso-cel) for the treatment of diffuse large B-cell lymphoma [3] and most recently Tecartus™ (brexucabtagene autoleucel) for the treatment of mantle-cell lymphoma in adults [4]. Multiple myeloma, which is considered a B-cell lymphoma by the WHO, is a malignant disease with uncontrolled proliferation of plasma cells in the bone marrow [5]. It is the second most frequent hematologic malignancy and remains a largely incurable disease [5]. Novel agents have been approved by the FDA, i.e., proteasome inhibitors (bortezomib, carfilzomib and ixazomib), immunomodulatory drugs (thalidomide, lenalidomide) and lately, monoclonal antibodies such as daratumumab and isatuximab against the CD38 receptor on multiple-myeloma cells and elotuzumab targeting SLAMF7 [6,7,8]. Due to this enrichment of therapeutical diversity, multiple-myeloma patients’ median survival duration improved from about 3 to 6 years [7]. In the search for a new target on multiple-myeloma cells for which to create suitable CAR-T cells, one has to look for an antigen that is highly expressed on the malignant target cells and very lowly expressed in healthy tissue [9,10]. BCMA largely fulfills these requirements. The 184-amino-acid-long transmembrane glycoprotein, which is part of the tumor-necrosis-factor receptor-family 17, regulates the proliferation, maturation and survival of plasma cells and is highly expressed in multiple-myeloma cells [11,12]. Several studies employing different CAR constructs have shown very promising results: Idecabtagene-vicleucel (ide-cel; Abecma™), a second-generation CAR construct comprising a CD28 co-stimulatory domain, yielded in a phase 1 study an overall response rate (ORR) of 76% and a complete remission (CR) rate of 39% [13], and in a subsequent phase 2 study similar values were confirmed [14]; ciltacabtagene-autoleucel (cilta-cel) had an ORR of 97% and a CR rate of 67% [15]; and orvacabtagene-autoleucel (orva-cel) had an ORR of more than 90% and a CR rate of around 40% [16]. In 2021, ide-cel became the first BCMA-directed CAR-T-cell therapy that was approved by the FDA and EMA. Even though initial response rates to ide-cel and other BCMA-targeting CAR-T-cell products are impressive, these responses are not durable for most patients as reflected by a median PFS of less than 10 months, as observed within the KarMMa trial [14], which clearly demonstrates the need for further improvement. Understanding CAR-T cell expansion and persistence kinetics is crucial for improving and establishing novel BCMA-targeting strategies.

**Figure 1:**
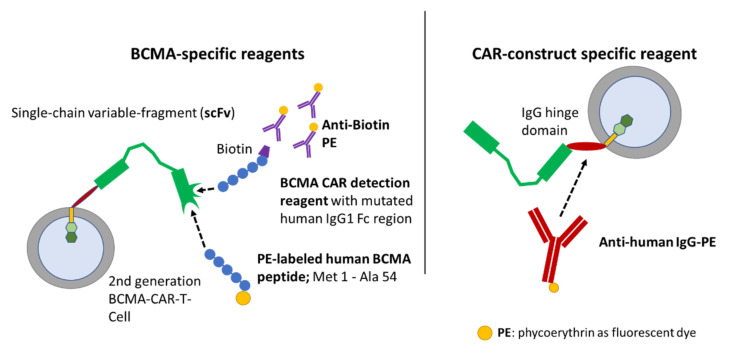
Binding mechanisms of flow-cytometric detection reagents. The left panel depicts BCMA-CAR-specific reagents. The right panel depicts a reagent which can be used for several different CAR constructs comprising a CH2-CH3-hinge region. The BCMA-detection reagent (Miltenyi Biotec) consists of a two-step staining mechanism: the first reagent is a fusion protein of the human BCMA extracellular domain and a mutated human IgG1 Fc region, therefore it does not require preliminary Fc blockage [17]. Moreover, this antigen-based detection reagent is conjugated to biotin. Therefore, the second component of the detection reagent is a monoclonal antibody directed against biotin and labeled with phycoerythrin (PE). Another specific staining approach employs a PE-labeled, truncated human BCMA protein/long peptide (ACRObiosystems), which consists of the BCMA protein amino-acid residues Met1 to Ala54. It is directly conjugated to a PE fluorochrome and therefore only requires a single staining step. The CAR-construct-specific reagent on the right can only bind to CAR constructs, which consist of a CH2-CH3-hinge region. The reagent consists of polyclonal-antibody serum directly conjugated to a PE fluorochrome.

Several methods for BCMA-CAR-T-cell quantification have been developed. However, a thorough comparison of these methods is pending. For this study we focused on three different BCMA-CAR-T-cell-detection reagents for flow cytometry as specified in Figure 1. Two of these assays relied on the binding of the CAR to BCMA-derived peptides, which were labeled directly (PE-labeled human BCMA peptide; ACRObiosystems, Beijing, China) or indirectly (BCMA-detection reagent; Miltenyi Biotec, Bergisch Gladbach, Germany) with fluorochromes. The third detection reagent (anti-human IgG PE; SouthernBiotech, Birmingham, AL, USA) employs a polyclonal-antibody serum directed against the CH2-CH3-hinge region, which is an integral part of several CAR constructs.

Moreover, a qPCR for BCMA-CAR-T-cell detection was established. Flow cytometry and qPCR, as the two most widely used assays for CAR-T-cell follow up, were compared. Therefore, a dilution series to evaluate the difference in the sensitivity of their ability to detect CAR-T cells was employed.

## 2. Results

### 2.1. Validation of Flow Cytometric BCMA-CAR Staining with Different Reagents

**Figure 2:**
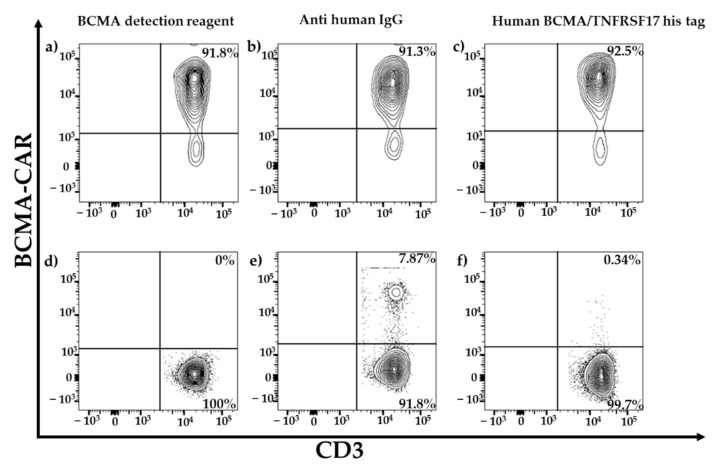
Comparison of different staining reagents for BCMA-CAR-T cells. Panels (**a**–**c**) show the transduction efficiency of BCMA-CAR-T cells manufactured from one healthy donor for the three reagents for staining BCMA-CAR-T cells in flow-cytometry contour plots. Panels (**d**–**f**) show the comparison of the specificity (background staining) of the three different reagents in flow-cytometry contour plots with outliners performed on fresh PBMCs from one healthy donor.

To compare different flow-cytometric BCMA-CAR-T-cell-detection methods, we used PBMCs (starting material), and BCMA-CAR-T cells manufactured from three different healthy donors (HD) HD1–HD3. Figure 2a–c show the transduction efficiencies of CD3^+^ T cells as an example of one HD fourteen days after transduction with the BCMA-CAR vector. All reagents were able to detect BCMA-CAR-T cells at a similar level and it was possible to clearly distinguish between BCMA-CAR-positive and negative T cells.

**Figure 3:**
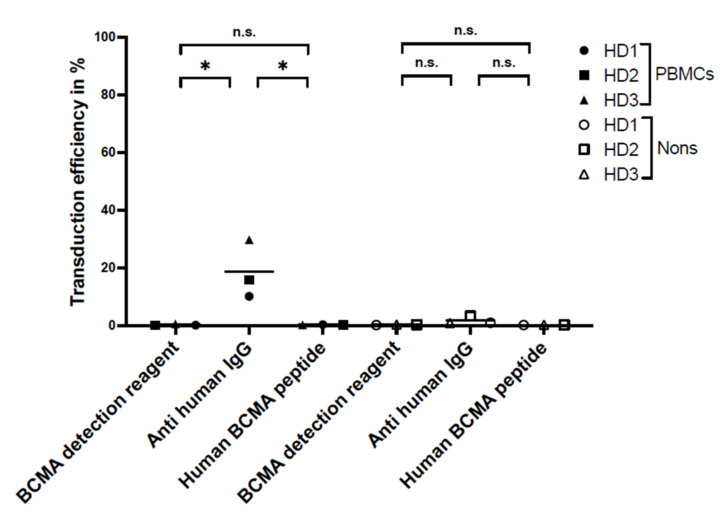
Specificity, i.e., background staining of non-transduced T cells from BCMA-CAR-T-cell manufacturing runs and fresh PBMCs of three HDs. Three staining reagents for the flow-cytometric analysis of transduction efficiency were compared, each one used on PBMCs and non-transduced T cells (Nons) (n.s. > 0.05, no significance, * *p* < 0.05, significant difference). The plot shows the mean values and statistical analysis with unpaired *t*-test.

Panels d–f of Figure 2 show an example of the background staining of fresh PBMCs from one healthy donor. To compare the specificity of the different reagents, we measured the false-positive staining on non-transduced T cells from BCMA-CAR-T-cell manufacturing runs and fresh PBMCs. Specificity showed significant differences between the reagents for the background staining of PBMCs (Figure 3). The PE-labeled human BCMA peptide showed a false-positive staining of 0.25 ± 0.06%. The polyclonal anti-human IgG PE antibody had a background of 7.2 ± 9.2%, and the BCMA-detection reagent had a very low false-positive staining of 0.04 ± 0.02%.

**Figure 4:**
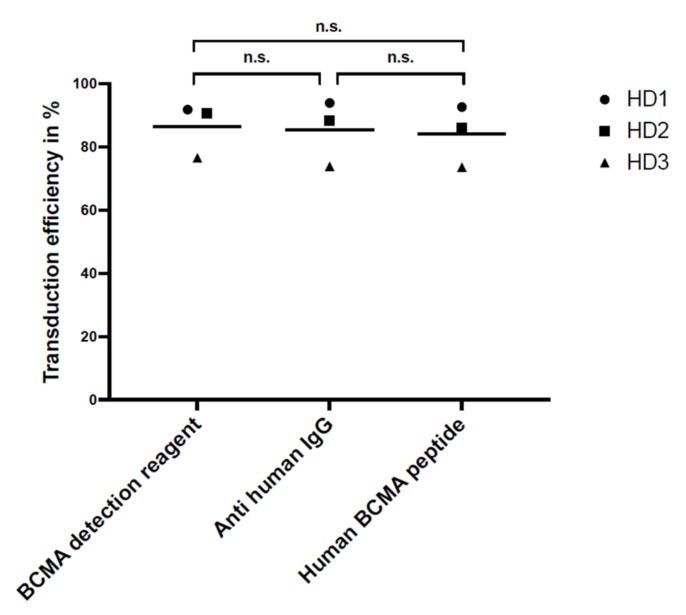
Sensitivity, i.e., transduction efficiency of CAR-T cells prepared from PBMCs of three different HDs using three different detection reagents measured by flow cytometry. There was no significant difference in sensitivity for the three reagents (n.s. > 0.05, no significance). The plot shows the mean values and statistical analysis with unpaired *t*-test.

The sensitivity, i.e., the ability to detect transduced BCMA-CAR-T cells from three BCMA-CAR-T-cell manufacturing runs that were performed with HDs stained with the three different reagents is shown in Figure 4. There were no significant differences between the sensitivity of the three reagents (*p* > 0.05).

### 2.2. Use of qPCR to Detect BCMA-CAR-T Cells

**Figure 5:**
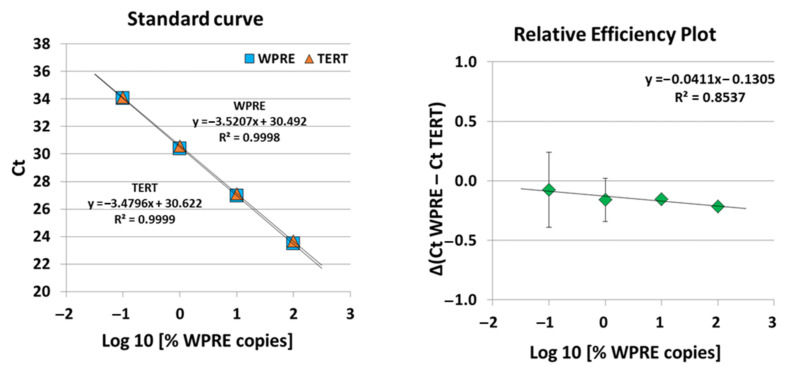
Standard curves and relative-efficiency plot of duplex qPCR reactions targeting WPRE and TERT in H_2_O standards (semi-logarithmic display; *x*-axis: % WPRE copies (log 10); *y*-axis: threshold cycle (Ct)). Exemplary data from one validation experiment are displayed. Mean Ct values from qPCR were used for linear regression. Reactions were performed in triplicate. qPCR: quantitative polymerase chain reaction. The relative-efficiency plot compares simultaneous PCR reactions over the tested Ct range by calculation of Δ(Ct WPRE − Ct TERT) and the use of graphical analysis (semi-logarithmic display; *x*-axis: % WPRE copies (log10); *y*-axis: ΔCt). Exemplary data from one validation experiment are shown. Mean Ct values from qPCR were used for ΔCt calculations. Results are represented as mean ± standard deviation (SD). Reactions were performed in triplicate.

In addition to flow-cytometric staining, we also evaluated the suitability of a qPCR method for the detection of BCMA-CAR-T cells. A detailed description of the qPCR validation strategy is provided in Section 4. For the assessed qPCRs, the efficiencies and linearities of the standard curves (H_2_O dilutions) were within accepted ranges for all validation runs. PCRs targeting the woodchuck hepatitis virus post-transcriptional regulatory element (WPRE; domain within the viral BCMA-CAR vector insert) and the telomerase reverse transcriptase (TERT; single copy gene) in H_2_O-standard samples displayed efficiencies of 94.6% and 95.4% with a similar standard deviation of ±3.2%. For all validation experiments, the relative efficiencies were very similar for defined target-concentration ranges. Exemplary data from one of the three validation experiments are displayed in Figure 5.

For recovery testing, genomic standards were generated by the serial dilution of genomic DNA from CAR-T cells into genomic DNA of non-transduced PBMCs of the same donor and quantified by qPCR. Table 1 summarizes the results of four experimental runs (EXP-1–EXP-4). Here, we observed a slowly increasing over-representation of the WPRE signal in the serial-diluted samples by assessment of the mean recovery per dilution (50–0.8%). Thus, we introduced a correction parameter which increases with the Δ(Ct WPRE − Ct TERT) and is only applied when Ct WPRE > Ct TERT for all future experiments with this specific qPCR. Assessed copy numbers are corrected by the following formula:

$$\frac{copy\;number\;per\;µg\;PBMC\;DNA}{1.08^{\;\Delta \left(\mathrm{Ct}\;\mathrm{WPRE}\;-\;\mathrm{Ct}\;\mathrm{TERT}\right)}}$$

The correction parameter is based on the efficiency of the WPRE-targeting PCR from the serial dilutions of the recovery experiment. Similar to Figure 5, but exclusively for the WPRE-targeting qPCR, the Log10 of the relative WPRE copies in terms of percent (*x*-axis) were graphically analyzed against the Ct (*y*-axis) (data not shown). The mean efficiency of the WPRE-specific PCR was increased to 108 ± 4.7% in these experiments with a stable TERT-specific PCR signal in every dilution. The defined correction parameter from above is based on the false increased amplification of 8% which is potentiated per cycle.

In terms of specificity for the WPRE-specific PCR, we observed an unspecific signal in some negative-control samples (non-transduced cells) from Ct 35.8 and higher. Thus, we set the technical limit of detection to Ct 35 for the WPRE-targeting PCR and samples with a Ct ≥ 35 were excluded from analysis. All relevant quantification parameters were defined as:

The measured upper limit of quantification (ULOQ) is ~575,617 copies/µg PBMC DNA. The lower limit of quantification (LLOQ) is ~1000 copies/µg PBMC DNA (~0.2% CAR-T cells). The lower limit of detection (LLOD) is ~100 copies/µg PBMC DNA (<0.05% CAR-T cells).

### 2.3. Cell Titration of BCMA-CAR-T Cells for qPCR vs. FACS Staining

**Figure 6:**
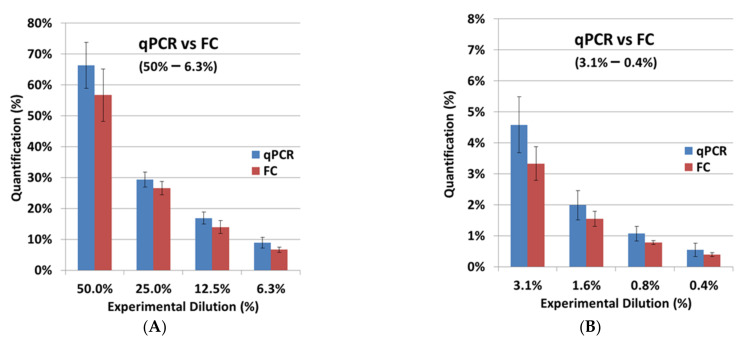
Comparison of quantification via qPCR with flow cytometry (FC). Graphical analysis of data from Table 2 (experimental dilution range of 50–0.4%). BCMA-CAR-T-cells were serially diluted 1:1 into non-transduced T cells of the same donor. A portion of each cell sample was used for extraction of genomic DNA and subsequent qPCR measurement. Another portion was applied to staining and flow cytometry. (**A**) Experimental dilution ranges from 50 to 6.3%. (**B**) Experimental dilution ranges from 3.1 to 0.4%. Three independent experiments were performed with cell material from different healthy donors (HD-1, HD-2, HD-3); bars with mean ± standard deviation (SD) are shown.

All the quantification results from qPCR compared to FACS are displayed in Table 2. The experiments were performed on cell material from three healthy donors (HD1–HD3). As shown in Figure 6, qPCR as well as FACS could reliably detect BCMA-CAR-T cells. Flow cytometry could detect BCMA-CAR-T cells to a minimum concentration of 0.4%; qPCR could detect even lower concentrations (0.02–0.01%). The quantified values for the experimental dilutions from 25% and below were normalized to the 50% sample and not the 100% stock sample due to over-representation in values when normalizing to the 100% sample for qPCR and FACS; the quantified values for the experimental dilution of 50% were normalized to the 100% stock sample.

## 3. Discussion

To improve therapy for patients with refractory multiple myeloma, BCMA-directed CAR-T-cell quantification and monitoring of CAR-T-cell expansion in peripheral blood is crucial. It is known that the response to CAR treatment is essentially influenced by the number of CARs in the patient’s body [18]. Moreover, it is important to know the number of CARs in the patient in order to differentiate the side effects of treatment from other causes such as infections [19].

In this study two widely used methods to detect CD19-CAR-T cells, flow cytometry and qPCR, were compared and established for the detection of BCMA-CAR-T cells.

For flow cytometry, the specificity and sensitivity of the staining reagents were differentiated, selecting the best reagent for the comparison study. Three different staining reagents were evaluated in a comparative study for BCMA-CAR detection. The PE-labeled human BCMA peptide as well as the BCMA-detection-reagent antibody have a specific binding to the single-chain Fv fragment of the BCMA-CAR. The polyclonal anti-human-IgG-antibody serum binds to the IgG1 CH2-CH3-hinge domain of the BCMA-CAR used in this study. There was no significant difference between the sensitivity of the reagents. Nevertheless, we found the BCMA-CAR-detection reagent and the PE-labeled human BCMA peptide to be the best suited to detect BCMA-CAR-T cells. They showed very high transduction efficiencies with a clear distinction for CAR-negative cells. Most importantly, they had the lowest background staining of all the reagents. This may be due to their specific targeting of the BCMA binding site on the CAR. The specific targeting of the BCMA domain, in contrast to the unspecific binding of the hinge region in other reagents, can also lead to a conclusion about the right folding of the CAR and its functionality. In contrast to the BCMA-detection reagent, which requires a two-step staining process, the PE-labeled human BCMA peptide requires only one single step. This can minimize experimental inaccuracy. Furthermore, due to fewer washing steps, fewer cells will be washed out, requiring lower amounts of cells. The polyclonal anti-human IgG antibody had the highest background staining, which may be due to the unspecific (not BCMA-specific) binding of the antibody.

A highly sensitive and specific qPCR assay, i.e., quantitative single-copy-gene (SCG)-based duplex (DP)-PCR assay (SCG-DP-PCR) for the quantification of our BCMA-CAR construct containing a vector insert was successfully validated. The duplex reactions of SCG-DP-PCR displayed similar efficiencies near 100% and high linearities (R^2^ > 0.98) (Figure 5). The PCR efficiencies were constant over the measured concentration range, i.e., there was a similar Δ(Ct WPRE − Ct TERT) in every H_2_O-standard sample, as confirmed by a relative-efficiency plot. Here, the log10 of relative copies in terms of percent (*x*-axis) were plotted against the Δ(Ct WPRE − Ct TERT) (y-axis). A slope of <0.1 (optimal 0) was obtained by applying linear regression, thereby proving constancy (Figure 5).

As a novelty in this work, we introduced a recovery assessment for SCG-DP-PCR and observed an efficiency increase to 108 ± 4.7% when the copy number of WPRE was decreased while TERT was kept stable. An efficiency of 100% would represent a complete doubling of the PCR-target per cycle and is theoretically the maximum possible for qPCR. However, standard-curve generation can mathematically result in values >100%. A value above 100% represents a false increase, which can have different reasons. For example, a PCR inhibitor in the highest concentrated standard results in a late Ct in this sample. The serial-diluted standards contain lower concentrations of this inhibitor, resulting in a more correct Ct in the diluted standards. An efficiency analysis in such a scenario results in an over-represented value >100%. We do not assume an inhibition effect for our recovery experiment because this would also affect the H_2_O standards mentioned above and in these experiments the efficiency was always <100%. Rather, we assume the error source to be the changed interference between parallel PCR runs due to the increasing difference in the target concentrations of the serial-diluted genomic standards compared to the rather similar relative concentrations of WPRE and TERT in the H_2_O standards. The TERT-targeting PCR in a high copy number starts at an early cycle, consumes dNTPs and leads to an exponential production of the TERT-specific amplicon and the corresponding fluorescence signal, in the diluted genomic standards. Whereas the WPRE-targeting PCR in a lower copy number starts at a late cycle and is exposed to a massive amount of the TERT amplicon and a decreased dNTP concentration. This imbalance in the reaction conditions for both PCRs could result in an error signal within the secondary induced PCR, i.e., WPRE in our setup. Furthermore, it is a possible reason for the observed slowly increasing over-representation of the WPRE signal and CAR quantification in the genomic standards by the assessment of the mean recovery per dilution (Table 1; 50–0.8%). We practically solved this issue by the introduction of a correction parameter described in the results Section 2.2.

Overall, our validated SCG-DP-PCR approach is advantageous compared to other qPCR approaches due to its high standardization by the independence of any reference samples, e.g., standards or calibrators. Such reference samples need to be produced repeatedly, thereby increasing the assay variance. The quantification by SCG-DP-PCR is only dependent on the PCR signals (WPRE and TERT) within the vector-insert-containing sample. Additional aspects of the applied qPCR strategy SCG-DP-PCR have been discussed in detail in our previous work from 2020 [20].

After the independent validation of both assays, FC and qPCR were compared. The FC results from the cell-titration experiment with BCMA-CAR-T cells were very similar to the experimental dilutions (Table 2; Figure 6). For SCG-DP-PCR, the previously defined correction parameter (Section 2.2) was applied to the quantification. Here we observed a minorly increased quantification in qPCR compared to FC. Our strategy to apply the viable cell count for the preparation of CAR-T-cell dilutions in non-transduced T cells was optimal for a direct cellular measurement, e.g., FC. Dead cells and aggregates were easily excluded by proper gating. QPCR, in contrast, with a molecular endpoint (DNA amplification) in the respective samples, is highly influenced by dead cells and aggregates, which are also quantified after DNA extraction. Potentially existing aggregates and varying death-cell amounts in the CAR-T-cell stock samples, which were used for the cell titration, could cause the increased quantification in qPCR. However, the observed difference of ~30% on average, e.g., resulting in a value of 1.3% for qPCR compared to 1% in FC, is acceptable. Depending on the copy number in the CAR-T-cell stock samples that were used for dilution, a qualitative specific detection was achieved down to minimum CAR-T-cell concentrations of 0.02–0.01% (1000–100 copies) with an applicable quantification ranging from 0.05–0.1% (≥1000 copies) (Table 2) using qPCR. Hence, the validated qPCR detects CAR-T cells with a 20-fold increase in sensitivity compared to the tested FC assay with a detection limit of 0.4%. However, FC is still very important to the application of a direct structural-endpoint measurement of the BCMA-CAR.

QPCR is already applied to the assessment of BCMA CAR-T-cell expansion in clinical phase studies [14,15]. In our work, we could additionally underline the suitability of qPCR for the assessment of BCMA-CAR-T-cell expansion by proving the comparability to FC in terms of the reflection of cellular kinetics. Thus, the molecular copy-number endpoint assessed by qPCR behaves similarly to a direct cellular endpoint in this context and can theoretically replace FC. The validated measuring range of our established qPCR (~100 copies/µg to ~575,617 copies/µg) is well appropriate for the future quantification of patient follow-up samples as the clinically relevant measuring range observed in BCMA-CAR-T-cell studies is largely enclosed. Clinically observed maximum expansions were up to ~100,000 for cilta-cel [15] and ~300,000–600,000 copies/µg for ide-cel [14]. Moreover, the lowest limit for a valid detection matches the clinically applied qPCR methods (~50–100 copies/µg). In addition, the measured ULOQ of our qPCR only reflects the highest concentrated stock sample quantified within our validation procedure and is no technical limit. We suppose that quantifications of two- to four-fold higher vector transgene levels than the measured ULOQ are possible.

Directly after the transfusion of CAR-T cells, as well as one month or later after transfusion, the number of CAR-T cells might be too low to use flow cytometry as a detection method. In these cases, qPCR might be more appropriate as it is the more sensitive method. The detection of CAR-T cells in the patients’ peripheral blood is an essential parameter to make the clinical decision on how to proceed with a particular patient: in the case of a low frequency of CAR-T cells, it might be a good option to stimulate and activate T cells by immunomodulatory agents such as lenalidomide in lymphoma patients or venetoclax or azacytidine in leukemia patients. In patients where CAR-T cells completely vanished, a second transplantation of CAR-T cells might be advisable. A persisting high frequency of CAR-T cells without any clinical effect can suggest a loss or splicing of the target antigen. In these patients, an approach with a different kind of CAR-T cell or an antibody targeting different antigens such as CD38 or SLAMF7 (e.g., daratumumab or elotuzumab) might bring the patient back into remission.

In conclusion, we recommend using a CAR-detection reagent with low background staining, like the BCMA-detection reagent. For monitoring BCMA-CAR-T cells in peripheral blood, both flow cytometry and qPCR are alternative methods. However, for very small concentrations of CAR-T cells, qPCR constitutes the most reliable method.

## 4. Materials and Methods

### 4.1. Isolation of PBMCs from Healthy Donors

For the isolation of PBMCs, buffy coats from the blood bank of the University Hospital of Mannheim were used with informed consent obtained from all patients. PBMCs were isolated through ficoll (Ficolite-H (Human), Linaris Biologische Produkte, Dossenheim, Germany) and cryopreserved in a nitrogen tank.

### 4.2. Manufacturing of BCMA-CAR-T Cells

CAR-T-cell production was performed following a protocol from Baylor College of Medicine, Houston, TX, USA. After activation of PBMCs with CD3/CD28 (Biolegend, San Diego, CA, USA) the cells were incubated at 37 °C and 5% CO2 for 24 h. Cells were then fed with IL-7 and IL-15 (R&D Systems, Minneapolis, MN, USA). The virus was incubated with retronectin (Takara Inc., Shiga, Japan) before transduction. We used a retroviral vector generated at the MDC-Berlin. For the construction of the second-generation CAR, a chimeric anti-human BCMA antibody J22.9-FSY was used [21]. The BCMA-CAR construct was composed of a Whitlow linker connecting the two-antibody, single-variable fragment chains (scFv). The scFv was fully humanized and was bound to the cell through a CH2-CH3-hinge region. Intracellular domains consisted of a CD28 co-stimulatory domain and a CD3zeta activation domain [8]. After 24 h incubation with the virus and refeeding, cells were harvested at day 10 and frozen in a nitrogen tank for 4 days.

### 4.3. Flow Cytometry CAR-Detection Validation

For the staining study with three different antibodies, cells were thawed and washed twice in PBS (Sigma-Aldrich, Munich, Germany). A quantity of 500,000 cells was used for each staining. The following antibodies were added to each experiment: 10 μL CD45 PerCP IVD (BD Biosciences, Franklin Lakes, NJ, USA), 10 μL CD3 FITC IVD (BD Biosciences) and to differentiate dead from viable cells the LIVE/DEAD Fixable Near-IR dead-cell stain kit (Thermo Fisher Scientific, Waltham, MA, USA) was used.

For staining the BCMA-CARs, the BCMA-detection reagent (Miltenyi Biotec, Bergisch-Gladbach, Germany) was used as a 1μL detection reagent with a 0.5μL anti-biotin. Cells were washed with 1 mL PBS and the BCMA-detection reagent was added and incubated for 10 min at room temperature. After two further washing steps, all other above-mentioned antibodies were added as well as anti-biotin PE and incubated for another 15 min at room temperature. After washing them with 1 mL FACS buffer (PBS/1% BSA/2 mM EDTA; Sigma Aldrich, St. Louis, MI, USA) cells were fixed in FACS fixing buffer (PBS/1% PFA/2 mM EDTA; Sigma Aldrich) and directly measured by flow cytometry.

For staining with the PE-labeled human BCMA peptide (ACRObiosystems, Newark, NJ, USA), the dilution was set to 1:50. Cells were washed with 1 mL PBS and the BCMA peptide was added with all other above-mentioned antibodies and incubated for 15 min at room temperature. After washing the cells with 1 mL FACS -buffer (PBS/1% BSA/2 mM EDTA; Sigma Aldrich) cells were fixed in FACS fixing buffer (PBS/1% PFA/2 mM EDTA; Sigma Aldrich) and directly measured by flow cytometry.

The polyclonal anti-human IgG PE antibody (SouthernBiotech, Birmingham, AL, USA) was diluted to 1:200. Cells were washed with 1 mL PBS and anti-human IgG PE was added and incubated for 10 min at room temperature. After two further washing steps, all other above-mentioned antibodies were added and incubated for 15 min at room temperature. After washing them with 1 mL FACS buffer (PBS/1% BSA/2 mM EDTA; Sigma Aldrich) cells were fixed in FACS fixing buffer (PBS/1% PFA/2 mM EDTA; Sigma Aldrich) and directly measured by flow cytometry.

For specificity testing, non-transduced BCMA-CAR-T cells and PBMCs of the three HDs were stained with the three above-mentioned detection reagents.

Compensation was performed with compensation beads (BD Biosciences) and amine-reactive compensation beads (Invitrogen, Waltham, MA, USA). Measurement was performed on FACS Canto (BD Biosciences) and analyzed by Diva v9.0 (BD Biosciences) and FlowJo v9 (BD biosciences) software.

### 4.4. qPCR for BCMA-CAR-T-Cell Quantification

General PCR conditions:

We extracted the genomic DNA from cell samples for qPCR analysis (cat. #51104, QIAamp DNA Blood Mini; QIAGEN, Hilden, Germany). For concentration measurements, UV spectroscopy was applied (NanoDrop OneC; Thermo Fisher Scientific, Applied Biosystems, Waltham, MA, USA). Samples were diluted to a final concentration of 20 ng/µL in nuclease-free H_2_O [20].

Thermal cycling for all PCR experiments was performed on the StepOnePlus real-time PCR system (Applied Biosystems) using the following amplification conditions: 50 °C for 2 min, 95 °C for 10 min, followed by 40 cycles of 15 s at 95 °C and 1 min at 60 °C. All PCR reactions contained two unlabeled primer pairs with a final concentration of 900 nM per primer and 250 nM for probes. The 2× TaqMan™ Universal PCR Master Mix (cat # 4304437) and each 20× primer probe mastermix (Custom TaqMan™ Gene Expression Assay, FAM, cat # 4331348 for the WPRE PCR and TERT Copy Number Reference Assay, cat # 4403316 for the TERT PCR) were purchased from Applied Biosystems. The final volume per PCR reaction was 25 μL containing 1.25 μL per 20× primer probe mastermix, 12.5 μL of 2× TaqMan™ Universal PCR master mix, 5 μL of sample (20 ng/μL) and 5 μL of nuclease free water. Non-template control (NTC) and a biological negative control (non-transduced donor cells) were included within all experiments. All reactions were performed in triplicate.

qPCR was performed using a novel approach, i.e., quantitative single-copy-gene (SCG)-based duplex (DP)-PCR assay (SCG-DP-PCR), which was introduced recently by our group [18,20]. Primers and a probe targeting specific sequences within the woodchuck hepatitis virus post-transcriptional regulatory element (WPRE) of the vector insert were generated using the primer-designing software Primer3Plus (https://www.bioinformatics.nl/cgi-bin/primer3plus/primer3plus.cgi, accessed on 10 October 2021). As a reference, the single-copy gene telomerase reverse transcriptase (TERT) was used from a commercially available kit.

In detail, the following primer sets were used for SCG-DP-PCR reactions:

(1) WPRE forward primer (FP): GCGTGGTGTGCTCTGTGTT, reverse primer (RP): AAAGGAGTTGACAGGTGGTG, probe: FAM-CTGACGCAACCCCCACTGG-MGB/NFQ. All oligonucleotides bind within the WPRE sequence of the integrated vector insert.

(2) TERT: Copy number reference assay, TERT (cat. #4403316, TaqMan; Applied Biosystems), containing TERT gene-specific forward primer, reverse primer, and probe (VIC/TAMRA) within a reaction mix.

Similar to our previous work [18], SCG-DP-PCR validation was based on the use of standard curves, without the need for these standards after successful validation. As the targeted sequences for this specific SCG-DP-PCR, WPRE and TERT, were used differently compared to our previous work [18,20], a separate validation was performed. For this, genomic DNA isolated from in vitro BCMA-CAR-T cells were used as the stock sample for standard preparation. The copy number of the CAR-T-cell stock was defined as 100%. H_2_O standards were generated via three serial 1:10 dilutions (100–0.1%) of the respective stock samples in nuclease-free H_2_O. Method validation and evaluation of SCG-DP-PCR was performed following the same strategy as already described [20]: using generated standard curves, the efficiencies (100 ± 10%) and linearities (correlation coefficient (R2) ≥ 0.98) of PCR reactions amplifying both targets within the duplex PCR (WPRE and TERT) were assessed. Constancy of PCR efficiencies across a wide target concentration range (0.1 to 100%) was tested, i.e., similarity of Δ (Ct WPRE − Ct TERT) in all standards via a relative-efficiency plot was confirmed. Three validation runs were performed.

After validation, SCG-DP-PCR was applied for recovery testing and the FACS comparison experiment, both of which are described below. Copy numbers were assessed using the 2^−ΔCt^ calculation as previously described [20], i.e., applying the formula:

$$\mathrm{copy}\;\mathrm{number}/µg\;\mathrm{PBMC}\;\mathrm{DNA}=2^{-\Delta \left(\mathrm{Ct}\;\mathrm{WPRE}\;-\;\mathrm{Ct}\;\mathrm{TERT}\right)}\times 2\times 140\;370$$

In addition to the described validation of SCG-DP-PCR, we performed an experiment for testing the recovery. Therefore, we generated genomic standards by serial dilution of genomic DNA extracted from in vitro BCMA-CAR-T cells into genomic DNA of non-transduced PBMCs. First, both stock samples (CAR-Ts and PBMCs) were prediluted with H_2_O to a similar concentration of 20 ng/µL. Subsequently, the CAR-T DNA was diluted via thirteen 1:1 dilutions (100–0.013%) into the PBMC genomic DNA. This resulted in a stable PCR signal of the reference gene TERT in all samples while the WPRE PCR signal serially decreased by experimental 1:1 dilutions. For all recovery calculations, the measured copy number in a genomic standard dilution was first normalized relative to the copy number in the stock sample. Then, the normalized values of each dilution were divided by the experimental dilution of the respective sample and converted into a percentage. An optimal recovery is indicated by a value of 100%, while decreased values under-represent and increased values over-represent the real quantity. In the case of an under- or over-representation, a correction parameter can be introduced into qPCR quantification.

### 4.5. Cell Titration of BCMA-CAR-T Cells for qPCR vs. FACS Staining

A vial containing 1 × 10^7^ BCMA-CAR-T cells and a vial containing 5 × 10^7^ non-transduced T cells were thawed in a 37 °C water bath. Then the cells were resuspended and transferred into a 50 mL round-bottom polystyrene tube (Falcon, Corning, Wiesbaden, Germany) containing 20 mL of warm PBS. Cells were filtered through a 70 μM cell strainer (Miltenyi Biotec). Two washing steps with PBS followed and then cells were counted using trypan blue (Biochrom, Berlin, Germany). BCMA-CAR-T cells and non-transduced cells were adjusted to a concentration of 2 × 10^6^/mL. Thirteen tubes were prepared with 1 mL of non-transduced cells. Then 1 mL of BCMA-CAR-T-cell suspension was added to tube 1, resuspended several times and was used for 12 subsequent serial-dilution steps in non-transduced PBMCs. In the end, we had samples in a dilution range from 50 to 0.0125% relative to the stock BCMA-CAR-T-cell sample and one stock of BCMA-CAR-T cells and non-transduced cells. For flow cytometry, 500 μL of the solution containing 1 × 10^6^ cells were stained directly after dilution. For qPCR, 500 μL of the solution containing 1 × 10^6^ cells were shock frozen in liquid nitrogen and then stored in a −80 °C freezer until further procedure.

For the qPCR vs. flow cytometry experiment, we used 1 × 10^6^ cells for each flow-cytometric analysis and 50,000 events were recorded. For staining of surface markers, CD45 PerCP IVD (Biolegend) and CD3 FITC IVD (Biolegend) were used. The LIVE/DEAD Fixable Near-IR dead-cell stain kit (Thermo Fisher Scientific) was used to differentiate dead from viable cells. Samples were measured on the LSRII flow cytometer (BD Biosciences) and analyzed by Diva (BD Biosciences) and FlowJo (BD Biosciences) software. Compensation was performed with compensation beads (BD Biosciences) and amine reactive compensation beads (Invitrogen).

For gating strategy in flow-cytometric analysis, dead cells were excluded by Near-IR. Duplets were excluded by FSC-H and FSC-A. Gating was effective for CD45^+^ single cells to exclude all cells but leukocytes. To focus on T-cell monocytes, natural killer cells and dendritic cells were excluded by gating only CD3-positive cells. CAR-positive cells were differentiated by gating only BCMA*PE- and CD3-positive cells using contour plots. For mathematical analysis of FACS results, we normalized the results by dividing the measured cell count by the experimental count.

SCG-DP-PCR was applied to genomic DNA extracted from the stock BCMA-CAR-T-cell sample and all cell dilutions with PBMCs that were used for FACS analysis. Copy numbers assessed with SCG-DP-PCR in every dilution were normalized relative to the copy number in the stock sample of produced BCMA-CAR-T cells for a comparison with the quantified FACS populations (%). The experiment was performed on PBMCs from three different donors.

### 4.6. Statistical Analysis

Data evaluation for statistical analysis was performed using Excel v16.0 (Microsoft, Redmond, WA, USA). For *p*-values the two-way *t* test was used and *p*-values below 0.05 were set as significant. For graphical analysis GraphPad Prism v9.1.0 (Graphpad Software Inc., San Diego, CA, USA) was used. If not explicitly mentioned, standard deviations SD (SD±) were calculated for the described results.

## Figures and Tables

**Table 1:** Recovery testing.

Experimental Dilution	Quantification				Recovery				Mean Recovery Per Dilution
	EXP-1	EXP-2	EXP-3	EXP-4	EXP-1	EXP-2	EXP-3	EXP-4	
50.0%	54.6%	51.3%	51.1%	50.4%	109.1%	102.6%	102.2%	100.7%	103.7 ± 3.6%
25.0%	28.7%	25.9%	26.5%	26.2%	114.7%	103.6%	105.8%	104.8%	107.2 ± 4.7%
12.5%	15.3%	14.5%	15.2%	14.3%	122.6%	116.0%	121.9%	114.1%	118.6 ± 3.6%
6.3%	8.7%	7.8%	8.2%	7.8%	138.6%	124.4%	131.1%	125.5%	129.9 ± 5.0%
3.1%	4.4%	4.1%	4.5%	4.2%	140.4%	130.1%	144.2%	133.0%	136.9 ± 4.8%
1.6%	2.1%	1.9%	2.2%	2.0%	132.7%	120.7%	141.9%	126.8%	130.5 ± 6.9%
0.8%	1.0%	1.1%	1.4%	1.1%	124.2%	135.5%	173.8%	135.7%	142.3 ± 15.2%
0.4%	0.5%	0.4%	0.7%	0.4%	124.8%	108.5%	173.0%	107.2%	128.4 ± 24.0%
0.2%	qualit.	0.2%	0.4%	0.2%	qualit.	100.9%	180.1%	119.0%	124.9 ± 30.3%
0.1%	qualit.	qualit.	0.15%	qualit.	qualit.	qualit.	157.7%	qualit.	---
0.05%	---	qualit.	qualit.	qualit.	---	qualit.	qualit.	qualit.	---

Experimental dilutions to 0.02% and 0.01% for all experimental runs and one sample of EXP-1 (0.05% dilution) were measured with a Ct above the defined detection limit of Ct ≥ 35 (or no Ct) and excluded from quantification (---). Qualit. means a valid qualitative signal which was below the lower limit of quantification (LLOQ) but above the lower limit of detection (LLOD) with a relatively high standard deviation (SD) amongst the replicates.

**Table 2:** qPCR and FACS results of genomic DNA from cell titration samples.

Experimental Dilution	qPCR Quantification			FACS Quantification		
	HD-1	HD-2	HD-3	HD-1	HD-2	HD-3
50.0%	59.6%	64.9%	74.3%	57.0%	48.0%	65.0%
25.0%	27.3%	28.7%	32.0%	28.1%	24.1%	27.4%
12.5%	15.4%	16.3%	19.0%	15.8%	11.8%	14.3%
6.3%	8.0%	7.8%	10.9%	7.1%	5.7%	7.1%
3.1%	4.1%	4.0%	5.6%	3.8%	2.7%	3.5%
1.6%	1.8%	1.6%	2.5%	1.7%	1.3%	1.7%
0.8%	0.9%	1.0%	1.3%	0.8%	0.7%	0.8%
0.4%	0.4%	0.4%	0.8%	0.5%	0.3%	0.4%
0.2%	0.2%	0.2%	0.4%	---	---	---
0.1%	0.1%	0.1%	0.1%	---	---	---
0.05%	qualit.	qualit.	0.08%	---	---	---
0.02%	qualit.	qualit.	qualit.	---	---	---
0.01%	---	---	qualit.	---	---	---

Experimental dilutions to 0.01% of HD-1 and HD-2 assessed by qPCR were measured with a Ct above the defined detection limit of Ct ≥ 35 (or no Ct) and excluded from quantification (---). Qualit. means a valid qualitative signal which was below the LLOQ but above the LLOD with a relatively high standard deviation (SD) in replicates. The lower detection limit for the flow-cytometry (FC) assay was approximately 0.4%. Dilutions to <0.4% were not detectable and excluded from flow-cytometry (FC) analysis (---).

## Data Availability

Not applicable.

## References

[1] Maude S.L., Frey N., Shaw P.A., Aplenc R., Barrett D.M., Bunin N.J., Chew A., Gonzalez V.E., Zheng Z., Lacey S.F. (2014). Chimeric antigen receptor T cells for sustained remissions in leukemia. N. Engl. J. Med..

[2] Neelapu S.S., Locke F.L., Bartlett N.L., Lekakis L.J., Miklos D.B., Jacobson C.A., Braunschweig I., Oluwole O.O., Siddiqi T., Lin Y. (2017). Axicabtagene Ciloleucel CAR T-Cell Therapy in Refractory Large B-Cell Lymphoma. N. Engl. J. Med..

[3] Teoh P.J., Chng W.J. (2021). CAR T-cell therapy in multiple myeloma: More room for improvement. Blood Cancer J..

[4] Shah B.D., Ghobadi A., Oluwole O.O., Logan A.C., Boissel N., Cassaday R.D., Leguay T., Bishop M.R., Topp M.S., Tzachanis D. (2021). KTE-X19 for relapsed or refractory adult B-cell acute lymphoblastic leukaemia: Phase 2 results of the single-arm, open-label, multicentre ZUMA-3 study. Lancet.

[5] Rollig C., Knop S., Bornhauser M. (2015). Multiple myeloma. Lancet.

[6] Martino M., Canale F.A., Alati C., Vincelli I.D., Moscato T., Porto G., Loteta B., Naso V., Mazza M., Nicolini F. (2021). CART-Cell Therapy: Recent Advances and New Evidence in Multiple Myeloma. Cancers.

[7] Mikkilineni L., Kochenderfer J.N. (2021). CAR T cell therapies for patients with multiple myeloma. Nat. Rev. Clin. Oncol..

[8] Bluhm J., Kieback E., Marino S.F., Oden F., Westermann J., Chmielewski M., Abken H., Uckert W., Hopken U.E., Rehm A. (2018). CAR T Cells with Enhanced Sensitivity to B Cell Maturation Antigen for the Targeting of B Cell Non-Hodgkin’s Lymphoma and Multiple Myeloma. Mol. Ther..

[9] Bruno B., Wasch R., Engelhardt M., Gay F., Giaccone L., D’Agostino M., Rodriguez-Lobato L.G., Danhof S., Gagelmann N., Kroger N. (2021). European Myeloma Network perspective on CAR T-Cell therapies for multiple myeloma. Haematologica.

[10] Abbott R.C., Cross R.S., Jenkins M.R. (2020). Finding the Keys to the CAR: Identifying Novel Target Antigens for T Cell Redirection Immunotherapies. Int. J. Mol. Sci..

[11] Yu B., Jiang T., Liu D. (2020). BCMA-targeted immunotherapy for multiple myeloma. J. Hematol. Oncol..

[12] Jasinski M., Basak G.W., Jedrzejczak W.W. (2021). Perspectives for the Use of CAR-T Cells for the Treatment of Multiple Myeloma. Front. Immunol..

[13] Munshi N.C., Larry D., Anderson J., Shah N., Jagannath S., Berdeja J.G., Lonial S., Raje N.S., Siegel D.S.D., Lin Y. (2020). Idecabtagene vicleucel (ide-cel; bb2121), a BCMA-targeted CAR T-cell therapy, in patients with relapsed and refractory multiple myeloma (RRMM): Initial KarMMa results. J. Clin. Oncol..

[14] Munshi N.C., Anderson L.D., Shah N., Madduri D., Berdeja J., Lonial S., Raje N., Lin Y., Siegel D., Oriol A. (2021). Idecabtagene Vicleucel in Relapsed and Refractory Multiple Myeloma. N. Engl. J. Med..

[15] Berdeja J.G., Madduri D., Usmani S.Z., Jakubowiak A., Agha M., Cohen A.D., Stewart A.K., Hari P., Htut M., Lesokhin A. (2021). Ciltacabtagene autoleucel, a B-cell maturation antigen-directed chimeric antigen receptor T-cell therapy in patients with relapsed or refractory multiple myeloma (CARTITUDE-1): A phase 1b/2 open-label study. Lancet.

[16] Mailankody S., Htut M., Lee K.P., Bensinger W., Devries T., Piasecki J., Ziyad S., Blake M., Byon J., Jakubowiak A. (2018). JCARH125, Anti-BCMA CAR T-cell Therapy for Relapsed/Refractory Multiple Myeloma: Initial Proof of Concept Results from a Phase 1/2 Multicenter Study (EVOLVE). Blood.

[17] Nimmerjahn F., Ravetch J.V. (2008). Fcgamma receptors as regulators of immune responses. Nat. Rev. Immunol..

[18] Schubert M.L., Kunz A., Schmitt A., Neuber B., Wang L., Huckelhoven-Krauss A., Langner S., Michels B., Wick A., Daniel V. (2020). Assessment of CAR T Cell Frequencies in Axicabtagene Ciloleucel and Tisagenlecleucel Patients Using Duplex Quantitative PCR. Cancers.

[19] Schubert M.L., Schmitt M., Wang L., Ramos C.A., Jordan K., Muller-Tidow C., Dreger P. (2021). Side-effect management of chimeric antigen receptor (CAR) T-cell therapy. Ann. Oncol..

[20] Kunz A., Gern U., Schmitt A., Neuber B., Wang L., Huckelhoven-Krauss A., Michels B., Hofmann S., Muller-Tidow C., Dreger P. (2020). Optimized Assessment of qPCR-Based Vector Copy Numbers as a Safety Parameter for GMP-Grade CAR T Cells and Monitoring of Frequency in Patients. Mol. Ther. Methods Clin. Dev..

[21] Oden F., Marino S.F., Brand J., Scheu S., Kriegel C., Olal D., Takvorian A., Westermann J., Yilmaz B., Hinz M. (2015). Potent anti-tumor response by targeting B cell maturation antigen (BCMA) in a mouse model of multiple myeloma. Mol. Oncol..

